# Age-Dependent Efficacy of Ezetimibe for Low-Density Lipoprotein Cholesterol Reduction in Japanese Patients with or without Type 2 Diabetes Mellitus

**DOI:** 10.3390/jcm9061675

**Published:** 2020-06-01

**Authors:** Satoshi Yamaguchi, Kageyuki Oba, Moritake Higa, Osamu Arasaki, Michio Shimabukuro

**Affiliations:** 1 Department of Diabetes, Endocrinology and Metabolism, School of Medicine, Fukushima Medical University, 1 Hikarigaoka, Fukushima 960-1247, Japan; mshimabukuro-ur@umin.ac.jp; 2 Department of Cardiology, Nakagami Hospital, 610 Noborikawa, Okinawa 904-2195, Japan; 3 Department of Cardiology, Tomishiro Central Hospital, 25 Ueta, Tomishiro, Okinawa 901-0243, Japan; koba@yuuai.or.jp (K.O.); oarasaki@yuuai.or.jp (O.A.); 4 Department of Diabetes and Life-Style Related Disease Center, Tomishiro Central Hospital, 25 Ueta, Tomishiro, Okinawa 901-0243, Japan; himoritake@yuuai.or.jp

**Keywords:** ezetimibe, low-density lipoprotein cholesterol, age

## Abstract

Ezetimibe reduces cardiovascular risk by lowering the levels of low-density lipoprotein cholesterol (LDL-C). However, there is limited information regarding the factors associated with ezetimibe-mediated LDL-C reduction. We investigated the factors associated with LDL-C reduction after ezetimibe administration in Japanese patients with or without type 2 diabetes mellitus (T2DM). This single-center retrospective observational study enrolled a total of 266 consecutive ezetimibe-naïve patients, of which 154 were excluded because of either switching from statin or fenofibrate to ezetimibe (*n* = 52) or ezetimibe discontinuation (*n* = 102). Finally, 112 patients were eligible for analysis. To identify the factors influencing LDL-C levels, univariate and multivariate linear regression analyses were performed after 52 weeks of ezetimibe treatment. Overall, advanced age, T2DM, and high baseline LDL-C were significantly associated with a greater decrease in LDL-C levels. In the non-T2DM group, advanced age and high baseline LDL-C were associated with greater decrease in LDL-C levels. In the T2DM group, baseline LDL-C was the only factor that influenced the change in LDL-C levels. Advanced age was significantly associated with higher LDL-C reduction in non-T2DM patients, but not in T2DM patients. Ezetimibe use might be beneficial in older patients without T2DM. The lack of association between age and the LDL-C lowering effect by ezetimibe in patients with T2DM may be due to yet unknown mechanism except low statistical power.

## 1. Introduction

Hyperlipidemia accelerates the progression of atherosclerosis and raises the risk of cardiovascular diseases [1]. Statin, an hydroxymethylglutaryl-CoA (HMG-CoA) reductase inhibitor, is irrefutably the first line pharmacological therapy that is used for lowering low-density lipoprotein cholesterol (LDL-C) levels [2,3] and reducing the risk of cardiovascular events [1,2,3,4]. Statin is the most widely used therapeutic agent for hyperlipidemia; however, some patients still exhibit significant residual cardiovascular risks [5]. A series of randomized control trials [6] have confirmed that aggressive LDL-C reduction is beneficial to combat residual cardiovascular risk. There is substantial variability among individuals with respect to response to statin [7]. Furthermore, statin dosing is not enough in cases where statin monotherapy does not achieve sufficient LDL-C lowering [8,9,10]. Ezetimibe is a selective cholesterol inhibitor that acts on the Niemann–Pick C1-like 1 protein, a cholesterol transport protein located at the brush border of the small intestine [11,12]. The combination of ezetimibe and statin can accomplish a more aggressive LDL-C reduction and can reduce further incidence of a cardiovascular event [10,11].

In Improved Reduction of Outcomes: Vytorin Efficacy International Trial (IMPROVE-IT) and other clinical trials [9,13,14,15], patients with type 2 diabetes mellitus (T2DM) were shown to exhibit a greater decrease in LDL-C when ezetimibe administered ezetimibe in addition to statin. Moreover, the combination of ezetimibe with statin enhanced the secondary prevention of cardiovascular events [9,16]. However, there is still limited information regarding the factors that influence the LDL-C lowering effect of ezetimibe, other than T2DM.

The present study aimed to examine the clinical factors that influence the change in LDL-C level after one year of ezetimibe treatment in patients with or without T2DM.

## 2. Materials and Methods

### 2.1. Participants

**Figure 1:**
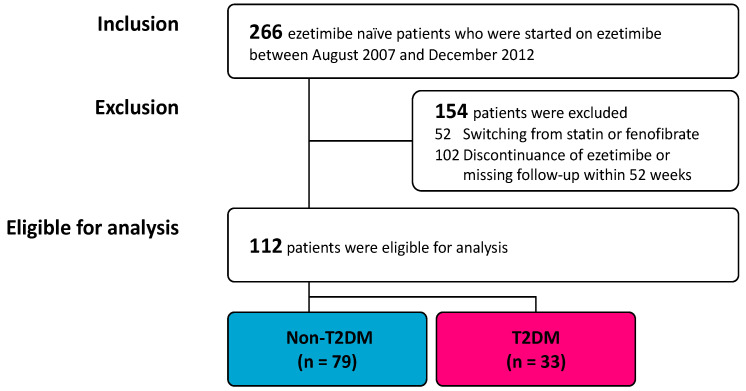
Flowchart of enrollment. T2DM, type 2 diabetes mellitus.

We conducted a single-center retrospective observational study. A total of 266 ezetimibe-naïve patients, who were started on ezetimibe treatment between August 2007 and December 2012, were included. Out of these patients, we excluded 154 patients because of the following reasons: switching from statin or fenofibrate to ezetimibe (*n* = 52) and discontinuation of ezetimibe within 52 weeks or missing from follow up during 52 weeks (*n* = 102). Finally, 112 patients were eligible for analysis (Figure 1). None of those patients had new-onset malignancy during the observational period. We divided the participants into two groups: patients with (T2DM, *n* = 33) and without T2DM (non-T2DM, *n* = 79).

When ezetimibe treatment was initiated, attending doctors and pharmacologists explained the purpose of ezetimibe and its usage. The registered nurse confirmed the patients’ compliance with the use of ezetimibe at every hospital visit.

The local ethical committee approved the present study. The present study adhered to the tenet of the Declaration of Helsinki. The need for informed consent was waived because of the observational nature of the study.

### 2.2. Data Collection

Blood samples were obtained from a brachial vein, in the fasting state, on the same day as ezetimibe administration was initiated and 52 weeks after the ezetimibe treatment. The analyses were performed immediately after blood sampling using Bio-Majesty JCA-BM 2250 (JEOL, Ltd., Tokyo, Japan). Medical technicians reanalyzed the data to check for out of reference values.

The primary outcome was the % change in LDL-C from baseline to follow up (52 weeks after the treatment). LDL-C levels were calculated using the Friedewald equation when the triglyceride (TG) level was equal to or less than 400 mg/dL, and were directly measured when the TG level exceeded 400 mg/dL.

We reviewed the medical chart to collect data on clinical characteristics, comorbidities (including hypertension and T2DM), coronary artery disease (defined as a patient with any of following: angina pectoris, organic coronary artery stenosis, old myocardial infarction, or history of percutaneous coronary intervention or coronary artery bypass grafting), chronic kidney disease (estimated glomerular filtration rate <60 mL/min/1.73 m^2^), antihyperlipidemic pretreatment (>4 weeks prior to start of ezetimibe administration), and laboratory data.

### 2.3. Statistical Analysis

Continuous variables with normal distribution and skewed distribution were expressed as mean ± standard deviation and median [25%, 75%], respectively. Categorical variables were expressed as a number with a percentage.

For the two-group comparison, the Student’s *t*-test and the Mann–Whitney U test were used for continuous variables with normal and skewed distribution, and the Fisher’s exact test was used for categorical variables. To evaluate the change in lipid profile including LDL-C, high-density lipoprotein cholesterol (HDL-C), and TG after 52 weeks of ezetimibe treatment, the Wilcoxon signed-rank test was used.

We examined age, gender, T2DM, hypertension, coronary artery disease, statin pretreatment, and baseline LDL-C of the patients using univariate linear regression analysis, to identify the factors associated with the change in LDL-C after ezetimibe treatment in the overall population and stratified them under T2DM or non-T2DM. The factors suggestive to be associated with LDL-C reduction in the univariate analysis (*p* < 0.10) were assessed using a multivariate linear regression model in the overall population and stratified under T2DM or non-T2DM.

A post hoc power calculation for the two-group comparison of % change in LDL-C between T2DM and non-T2DM was performed as described previously [17]. We also assessed the statistical power for the multivariate linear regression model to determine the factors associated with the efficacy of ezetimibe in each model [18].

The statistical software used to analyze the data included R 3.5.1 (R Project for Statistical Computing, Vienna, Austria) and Prism 8.0.0 (GraphPad Software, Inc., La Jolla, CA, USA). All statistical tests were two-tailed and *p* < 0.05 was considered to be statistically significant.

## 3. Results

### 3.1. Participants

In overall, the median age was 60 years. There were 64/112 (56%) male patients, of which 33/112 (29%) patients had T2DM. There were no significant differences in age, gender, body weight, and body mass index between the patients with and without T2DM. Hypertension, coronary artery disease, chronic kidney disease, and hemodialysis were more frequently seen in the T2DM group than in the non-T2DM group (Table 1; Figure S1).

### 3.2. Lipid Profile

**Figure 2:**
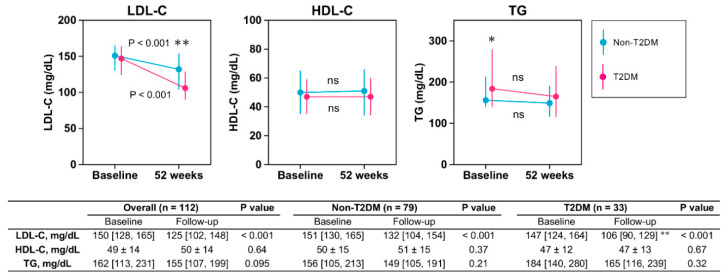
Lipid profiles before and after treatment of ezetimibe in non-T2DM and T2DM groups. Values are mean ± SD or median (25%, 75%). HDL-C, high-density lipoprotein cholesterol; LDL-C, low-density lipoprotein cholesterol; TG, triglyceride; T2DM, type 2 diabetes mellitus. * *p* < 0.05; ** *p* < 0.01 vs. non-T2DM. *p* values vs. baseline. ns: not significant.

**Figure 3:**
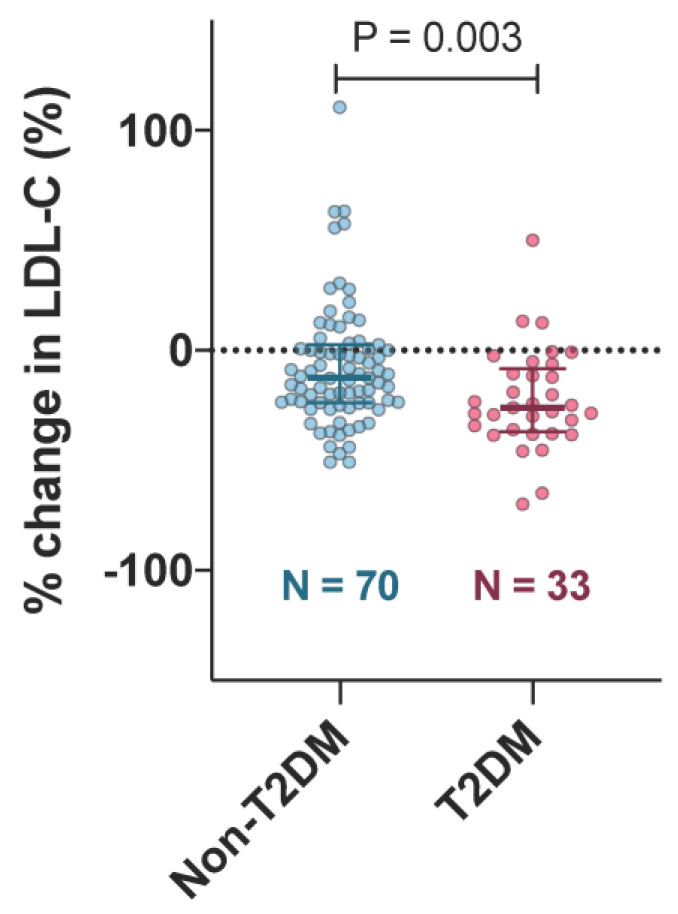
Comparison of % change in LDL-C level between non-T2DM and T2DM groups. LDL-C, low-density lipoprotein cholesterol; T2DM, type 2 diabetes mellitus. Bars represent median (25%, 75%).

There were no significant differences in baseline LDL-C and HDL-C between the non-T2DM and T2DM groups (Figure 2). Baseline TG in the T2DM group was higher than that in the non-T2DM group. Follow-up LDL-C in the T2DM group was significantly lower than that in the non-T2DM group (Figure 2). Percent change in LDL-C in the T2DM group was greater than that in the non-T2DM group (non-T2DM: −12.4 (−23.8 to 2.6) vs. T2DM: −26 (−37 to −8.4), *p* = 0.004; Figure 3).

### 3.3. Factors Associated with Reductions in LDL-C after Ezetimibe Treatment

**Figure 4:**
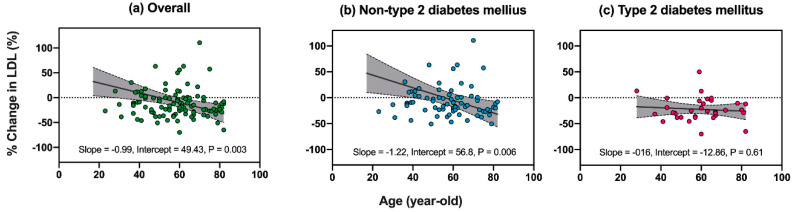
Correlations between age and % change in LDL-C. The Pearson correlation coefficient is used to calculate slope (solid lines) with 95% confidence interval (dotted lines).

In overall, univariate linear regression analysis revealed age and baseline LDL-C to be significant factors associated with the % change in LDL-C (age, coefficient = −0.99, *p* = 0.003, Figure 4a; baseline LDL-C, coefficient = −0.52, *p* < 0.001, Table 2). The association of LDL-C reduction with T2DM was not significant; however, a trend was observed (coefficient = −20.3, *p* = 0.056). In overall, multivariate linear regression analysis showed that age and baseline LDL-C were significantly associated with a reduction in LDL-C (age, coefficient = −0.75, *p* = 0.016; baseline LDL-C, coefficient = −0.48, *p* < 0.001; Table 2).

In the non-T2DM group, age and baseline LDL-C showed significant correlation with % change in LDL-C (age, coefficient = −1.22, *p* = 0.006, Table 2 and Figure 4b; baseline LDL-C, coefficient = −0.58, *p* < 0.001, Table 2). Similarly, the multivariate linear regression model showed that age and baseline LDL-C were significantly associated with the % change in LDL-C in this group (age, coefficient = −0.97, *p* = 0.024, Table 2; baseline LDL-C, coefficient = −0.5, *p* < 0.001, Table 2).

In the T2DM group, there was no significant correlation between age and the % change in LDL-C (*p* = 0.61; Figure 4c). The baseline LDL-C was the only significant factor that was associated with the LDL-C reduction (baseline LDL-C, coefficient = −0.39, *p* < 0.001; Table 2). Thus, multivariate linear regression analysis was not employed for this group.

### 3.4. Post Hoc Power Calculation

The estimated statistical power for the two-group comparison of the % change in LDL-C between the non-T2DM and T2DM groups was 0.96. The calculated statistical power of a univariate linear regression model to estimate % change in LDL-C using age in overall, non-T2DM and T2DM were 0.84, 0.81, and 0.09. The multivariate linear regression model to determine the factors that influence the LDL-C lowering effect of ezetimibe in the overall population and the non-T2DM group had 0.98 and 0.94 of statistical power.

## 4. Discussion

The present study compared the factors associated with ezetimibe-mediated LDL-C reduction in Japanese patients with or without T2DM. There were two major findings. First, the T2DM group exhibited a larger LDL-C reduction than the non-T2DM group (Figure 3). Second, the factors associated with ezetimibe-mediated LDL-C reduction were different in the patients with or without T2DM; a significant negative relationship between age and the % change in LDL-C was observed in the non-T2DM group (Figure 4b) but not in the T2DM group (Figure 4c). This study is the first to show that the effect of ezetimibe on LDL-C might be age-dependent in non-diabetic patients, but not in diabetic patients.

### 4.1. Factors Influencing the Efficacy of Ezetimibe

T2DM and high baseline LDL-C have previously been reported to be associated with the LDL-C lowering effects of ezetimibe [14,15]. Meanwhile, to determine the clinical factors that influence the LDL-C lowering effects of ezetimibe, Goldberg et al. [19] examined 1229 T2DM patients and found that older patients (≥65 years old) had a preferable lipid profile change, such as greater LDL-C decrease and high-density lipoprotein cholesterol (HDL-C) increase, compared to younger patients (<65 years old). The results of the current study showed that T2DM patients exhibited a greater LDL-C decrease than patients without T2DM after 52 weeks of ezetimibe treatment (Figure 3), which was consistent with previous reports [14]. In the patients without T2DM, ezetimibe administration led to a greater LDL-C reduction in older patients than in younger patients (Figure 4, Table 2). In contrast, the T2DM patients did not exhibit such age-dependent responses to ezetimibe (Figure 4, Table 2). Considering the result of both previous reports [19] and the current study, the lack of association between age and the LDL-C lowering effect by ezetimibe in patients with T2DM may be due to yet unknown mechanism. However, low statistical power might be partially responsible for the absence of an age-dependent effect of ezetimibe in patients with T2DM. Clinically, aging effects on ezetimibe efficacy may be limited in patients with T2DM.

The IMPROVE-IT trial [9,14], a large randomized clinical trial, demonstrated the effectiveness of ezetimibe administration as secondary prevention in patients with myocardial infarction. The clinical effectiveness in patients with T2DM has been reported in terms of a greater LDL-C decrease, which results in low risk of a cardiac event [20]. Therefore, ezetimibe should be recommended for T2DM patients regardless of their age. In the patients without T2DM, older patients might be considered as beneficial candidates for the ezetimibe-mediated LDL-C lowering treatment.

### 4.2. Limitations

The present study had several limitations. The patients’ compliance with the ezetimibe regimen was not assured, although a registered nurses confirmed their compliance. We could not address the clinical information that could modify the LDL-C lowering effect of ezetimibe, such as severity of T2DM, diet, and daily exercise. We could not follow up clinical events entirely such as cardiovascular events or new-onset malignancy. Despite the limitations of this study, we demonstrated the importance of age in patients without T2DM while considering cost-effectiveness of the ezetimibe treatment for hyperlipidemia. The lack of association between age and LDL-C lowering effect by ezetimibe in patients with T2DM might be due to low statistical power. Well-designed studies to further elucidate the factors that are associated with the response to ezetimibe are warranted.

## 5. Conclusions

A greater decrease in LDL-C levels was observed in the T2DM group than in the non-T2DM group. There was a significant negative correlation between age and change in LDL-C levels after 52 weeks of ezetimibe treatment in the overall population and in the non-T2DM patients. Advanced age is a significant factor for the LDL-C lowering effect of ezetimibe in non-T2DM patients, but not in T2DM patients. Ezetimibe should be recommended to patients with T2DM, although it might be most beneficial for older patients without T2DM.

## Figures and Tables

**Table 1:** Baseline clinical characteristics and lipid profile before and after ezetimibe treatment.

	Overall	Non-T2DM	T2DM	*p* Value
	*n* = 112	*n* = 79	*n* = 33	
Age, years	60 (49, 67)	59 (49, 68)	60 (50, 67)	0.5
Male gender, *n* (%)	64/112 (56)	41/79 (52)	22/33 (67)	0.21
Body weight, kg	66 (57, 74)	67 (55, 72)	65 (57, 76)	0.6
Body mass index, kg/m^2^	25.5 (23.4, 28.1)	25.4 (22.9, 28)	25.9 (23.8, 28.1)	0.61
Past medical history, *n* (%)				
Hypertension	46/112 (41)	20/79 (25)	26/33 (79)	<0.001
Coronary artery disease	15/112 (13)	4/79 (5.1)	11/33 (33)	<0.001
Chronic kidney disease	26/112 (23)	14/79 (18)	12/33 (36)	<0.001
Hemodialysis	16/112 (14)	4/79 (5.1)	12/33 (36)	<0.001

T2DM, type 2 diabetes mellitus.

**Table 2:** Univariate and multivariate linear regression analyses to identify factors associated with% change in LDL-C.

**Overall (*n* = 112)**								
	**Univariate**				**Multivariate**			
					**Adj R^2^ = 0.19, *p* < 0.001**			
	**Coefficient**	**95% CI**		***p* Value**	**Coefficient**	**95% CI**		***p* Value**
Age, 1 year	−0.99	−1.64	−0.34	0.003	−0.75	−1.37	−0.14	0.016
Male gender	−12.94	−32.07	6.19	0.19				
Type 2 diabetes mellitus	−20.3	−40.93	0.34	0.056	−14.51	−36.22	7.19	0.19
Hypertension	−17.36	−36.53	1.81	0.079	−10.45	−30.5	9.6	0.31
Coronary artery disease	−11.06	−39.06	16.95	0.44				
Statin pretreatment	5.36	−15.25	25.97	0.61				
Baseline LDL-C, mg/dL	−0.52	−0.77	−0.27	<0.001	−0.48	−0.72	−0.24	<0.001
**Non-Type 2 Diabetes Mellitus (*n* = 79)**								
	**Univariate**				**Multivariate**			
					**Adj R^2^ = 0.17, *p* < 0.001**			
	**Coefficient**			***p* Value**	**Coefficient**	**95% CI**		***p* Value**
Age, 1 year	−1.22	−2.07	−0.37	0.006	−0.97	−1.79	−0.14	0.024
Male gender	−11.97	−37.82	13.88	0.37				
Hypertension	−14.20	−43.89	15.49	0.35				
Coronary artery disease	−0.17	−59.39	59.06	>0.99				
Statin pretreatment	13.1	−14.98	41.18	0.36				
Baseline LDL-C, mg/dL	−0.58	−0.9	−0.25	<0.001	−0.5	−0.82	−0.18	0.003
**Type 2 Diabetes mellitus (*n* = 33)**								
	**Univariate**				**Multivariate**			
	**Coefficient**			***p* Value**				
Age, 1 year	−0.16	−0.76	0.44	0.61				
Male gender	−6.92	−23.6	9.76	0.42				
Hypertension	−1.07	−20.51	18.36	0.91	Univariate linear regression models did not reveal any significant factor			
Coronary artery disease	−1.29	−18.14	15.57	0.88				
Statin pretreatment	−10.37	−26.83	6.09	0.23				
Baseline LDL-C, mg/dL	−0.39	−0.59	−0.18	<0.001				

CI, confidence interval; LDL-C, low-density lipoprotein cholesterol.

## Supplementary Materials

The following are available online at https://www.mdpi.com/2077-0383/9/6/1675/s1, Figure S1: The comparisons of baseline characteristics in non-T2DM and T2DM groups.
