# Supplementary material for: Age-Dependent Efficacy of Ezetimibe for Low-Density Lipoprotein Cholesterol Reduction in Japanese Patients with or without Type 2 Diabetes Mellitus
Source: J Clin Med. 2020 Jun 1;9(6):1675. doi: 10.3390/jcm9061675 (PMC7356893; doi:10.3390/jcm9061675)
Supplement: Supplementary file 1 [file jcm-09-01675-s001.pdf]

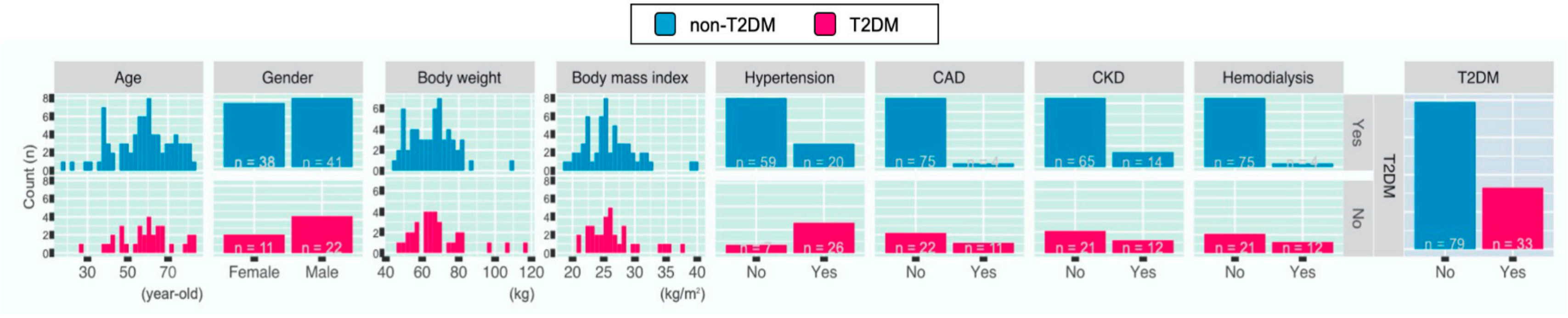

Figure 1. The comparisons of baseline characteristics in non-T2DM and T2DM groups. CAD, coronary artery disease,: CKD, chronic kidney disease; T2DM, type 2 diabetes mellitus; In continuous variables (age, body weight, and body mass index) , histograms show the distributions of variables. In categorical variables (gender, hypertension, CAD, CKD, hemodialysis and T2DM), bar graphs represent the numbers of patients.
